# Evaluation of standard treatments for managing adult Japanese patients with inadequately controlled moderate‐to‐severe atopic dermatitis: Two‐year data from the ADDRESS‐J disease registry

**DOI:** 10.1111/1346-8138.16485

**Published:** 2022-06-17

**Authors:** Norito Katoh, Hidehisa Saeki, Yoko Kataoka, Takafumi Etoh, Satoshi Teramukai, Hiroki Takagi, Hiroyuki Fujita, Marius Ardeleanu, Elena Rizova, Kazuhiko Arima, the ADDRESS‐J Investigators

**Affiliations:** ^1^ Department of Dermatology Kyoto Prefectural University of Medicine Graduate School of Medical Science Kyoto Japan; ^2^ Department of Dermatology Nippon Medical School Tokyo Japan; ^3^ Department of Dermatology Osaka Habikino Medical Center Osaka Japan; ^4^ Atago Dermatology Clinic Tokyo Japan; ^5^ Department of Dermatology Tokyo Teishin Hospital Tokyo Japan; ^6^ Department of Biostatistics Kyoto Prefectural University of Medicine Kyoto Japan; ^7^ Sanofi K.K. Tokyo Japan; ^8^ Regeneron Pharmaceuticals, Inc. Tarrytown New York USA; ^9^ Sanofi Cambridge Massachusetts USA

**Keywords:** adults, atopic dermatitis, flares, long‐term treatment, real‐world study

## Abstract

Atopic dermatitis (AD), a chronic relapsing inflammatory skin disease with a high disease burden, is one of the most common dermatological conditions in Japan. Herein, we report the disease profiles and current AD treatment during 2‐year management of Japanese adults with moderate‐to‐severe AD. ADDRESS‐J was a prospective, longitudinal, observational study that evaluated real‐world effectiveness and safety of current AD treatments of adult patients with moderate‐to‐severe AD (Investigator's Global Assessment score 3 or 4) in Japan. The maximum follow‐up period was 2 years. Among 300 patients enrolled, 288 had ≥1 post‐baseline evaluation and were analyzed (mean age, 35.5 years; 60.1% male). Almost all patients (99.7%) received topical therapy; the most commonly used therapy was topical corticosteroids of the second‐highest potency (86.5%) (e.g., 0.1% mometasone furoate) followed by medium‐potency topical corticosteroids (50.3%) (e.g., 0.05% clobetasol butyrate). At month 12 of the study, 10.4% of patients had Investigator's Global Assessment 0/1, similarly at month 24 (10.8%). A total of 132 patients (45.8%) had ≥1 AD flare‐up during the observation period, with the majority of first flares occurring within the first year of the study. Various physician‐ and patient‐reported outcomes improved considerably during the first 3 months of the study, with only minor changes after this time. In this cohort, 16.7% of patients had skin infections requiring treatment; 7.3% had adverse events (AE) potentially related to treatment; 1.7% had serious AE; and 1.0% had treatment discontinuations due to AE. Limitations include missing data at later timepoints and the inclusion criteria limiting generalizability. In summary, this analysis of the ADDRESS‐J study showed that some patients with moderate or severe AD respond to conventional therapies, while others do not. For those with inadequately controlled moderate‐to‐severe AD, the newly emerged systemic agents, such as biologics, may provide a potential strategy for long‐term disease management.

## 1 INTRODUCTION

Atopic dermatitis (AD), a chronic relapsing inflammatory skin disease with a high disease burden, is one of the most common dermatological conditions in Japan.^1^, ^2^

The point prevalence of AD was estimated at 6.9% in Japanese adults in 2004.^3^ Among adult patients with AD, the prevalence of moderate‐to‐very severe AD was estimated to be 23.3% (moderate, 18.5%; severe, 3.4%; very severe, 1.4%); around 20% of the patients had AD that was poorly controlled by topical therapies, including topical corticosteroids (TCS).^4^

According to the current treatment guidelines, the standard treatments for AD in Japan include TCS and topical calcineurin inhibitors (TCI) for controlling inflammation, emollients for skin care, and adjunctive therapies such as antihistamines for reducing pruritus; furthermore, treatment for severe refractory disease only includes higher‐potency TCS or TCS in combination with phototherapy, cyclosporine, or psychosomatic therapy.^5^ However, a substantial proportion of Japanese patients with AD suffer from inadequate disease control due to insufficient therapeutic response to TCS and TCI, poor treatment compliance attributable to self‐perceived improvement of disease, forgetfulness, and treatment inconvenience.^6^, ^7^

Thus far, several retrospective analyses or chart reviews have been performed to determine the long‐term outcomes in adult Japanese patients with AD.^4^, ^8^ Due to the emergence of novel therapies,^9^ the current real‐life practices for the long‐term management of AD should be assessed prior to positioning new treatment modalities.

To better understand the status of current AD treatments and longer‐term outcomes of available standard AD therapies in Japan, large‐scale, prospective, multicenter studies are needed. Here, we report such outcomes during the 2‐year management of adult Japanese patients with moderate‐to‐severe AD enrolled in the Japan AD Registry ADDRESS‐J.

## 2 METHODS

### 2.1 Study design

ADDRESS‐J (UMIN‐CTR: UMIN000022623) was a prospective, longitudinal, observational study aimed at evaluating real‐world effectiveness and safety of current treatments of moderate‐to‐severe AD in adult patients; a detailed study design has been reported previously.^10^ The total maximum follow‐up period for each patient was 2 years. The first patient had their first visit on 29 July 2016, and the last patient completed the study on 12 July 2019.

Treatment was prescribed according to the physician's clinical judgment or per standard of care for AD. Evaluations of the current treatment, including measures of its effectiveness and safety were carried out approximately every 3 months.

This study was conducted at 30 medical institutions in Japan in accordance with ethical principles that derive from the Declaration of Helsinki (1964) and all subsequent amendments as well as “Ethical Guidelines for Medical and Health Research Involving Human Subjects” (established on 22 December 2014).^11^ The protocol and other necessary documents were submitted and approved by the Institutional Review Board/Ethics Committees. Written informed consent was obtained from all patients prior to commencement of any study procedure.

### 2.2 Patients

Patients were included in this registry if they were Japanese males or females aged ≥20 to <60 years, with a diagnosis of AD according to the “Definition and diagnosis criteria for atopic dermatitis” specified by the Japanese Dermatological Association (JDA)^5^ ≥6 months before the baseline visit. They also had to have an Investigator's Global Assessment (IGA) score ≥3 (moderate or severe AD), suggesting inadequate control with their current treatment, and have treatment escalation at the baseline visit. Treatment escalation included starting any of the following treatments: a new AD medication (topical/oral corticosteroids or topical/oral immunosuppressants); change to a higher‐potency class of TCS; or an increase of AD medication dosage (topical/oral corticosteroids or topical/oral immunosuppressants). For the purpose of this study, ultraviolet (UV) phototherapy or antihistamine/anti‐allergic drugs were not considered as an escalation of treatment at baseline. Thorough implementation of the standard of care for AD (topical/oral corticosteroids or topical/oral immunosuppressants) such as patient education (brief instructions on treatment usually provided on a routine outpatient basis were not regarded as patient education) was also regarded as an escalation of treatment. Patients were excluded if they received treatment with an investigational drug within 8 weeks before the baseline visit or if they had any skin comorbidities that may have interfered with study assessments.

All patients who met the above inclusion criteria and were willing and able to comply with specified study‐related procedures (including understanding and answering various questionnaires) for 2 years and provided signed informed consent were included until the target enrollment of 300 patients had been met.

### 2.3 Outcomes

The main endpoints of the study were assessment of effectiveness and safety of current AD treatments. Efficacy measures included incidence of flares and percentage of patients with IGA status 0 (clear), 1 (almost clear), 2 (mild), 3 (moderate), or 4 (severe) during the study period. In this study, a flare was defined as worsening of AD symptoms with IGA score ≥3 requiring escalation of AD treatment per the investigator's judgment (optimization of the standard of care for AD, addition/dose increase of AD medication, or change to a higher‐potency class of TCS). Safety was assessed by the investigator and only included adverse events (AE) potentially related to AD treatment (causality assessed by the investigator); AE leading to AD treatment discontinuation; skin infection AE requiring treatment; and serious AE. AE were coded using Preferred Term and System Organ Class using the Medical Dictionary for Regulatory Activities/Japanese (MedDRA/J) version 22.0. In this study, worsening of AD itself was not regarded as an AE.

Other endpoints included change in Eczema Area and Severity Index (EASI);^12^ percentage of body surface area (BSA) affected by AD; as well as patient‐reported outcomes (PRO) such as Peak Pruritus Numerical Rating Scale (NRS) score in the past 7 days,^13^, ^14^ Dermatology Life Quality Index (DLQI),^15^ and Patient‐Oriented Eczema Measure (POEM).^16^ The following biomarkers were assessed if available: serum thymus‐ and activation‐regulated chemokine (TARC) levels, peripheral blood eosinophil count, serum total immunoglobulin E (IgE) levels, and serum lactate dehydrogenase (LDH) levels.^5^

### 2.4 Analysis

Sample size calculations showed that 270 patients allowed for the average incidence rate of flares within the 2‐year observation period to be estimated with a width of 0.2 flares/patient‐year 95% confidence interval (CI) assuming 1.5 flares/year in a patient. Considering a 10% dropout rate, the total number of patients was set as 300. The target sample size of 300 patients could also detect AE occurring with a frequency of approximately ≥1% with 95% probability.^17^

The average incidence rate of flares (unit: number of events/patient‐year) with 95% CI were estimated from number of flares and patient‐years at risk for every 3 months and over the 2‐year observation period. A Kaplan–Meier analysis of time to first flare occurrence was also conducted.

Other measures of treatment effectiveness were summarized every 3 months and at unscheduled visits. The window for the 3‐month data point was from the day after baseline visit and up to 4.5 months; for subsequent data points, this window was from 1.5 months before to 1.5 months after the pre‐specified time point. To describe the potential differential therapeutic impact of baseline medications, longitudinal scores for these measures were analyzed by use of baseline medications such as topical medications only, oral non‐steroidal systemic immunosuppressants, oral corticosteroids, and phototherapy.

All analyses were carried out with the SAS statistical package version 9.2 or later (SAS Institute, NC, US).

### 2.5 Sites and investigators

Patients were enrolled at 30 sites: 20 general dermatology clinics and 10 hospitals with ≥20 beds (of which eight were university hospitals). Most of the principal investigators were JDA‐certified dermatologists (28/30; 93.3%).

## 3 RESULTS

### 3.1 Patients

A total of 300 patients were enrolled between 29 July 2016 (first patient in) and 4 July 2017 (last patient in); 12 patients were excluded because they did not attend any post‐baseline visits and did not answer any of the questionnaires for PRO. The efficacy analysis set and the safety analysis set were identical, and both comprised 288 patients.

Of the 300 patients enrolled, 54 discontinued from the study. Reasons for discontinuation included consent withdrawal (*n* = 26), loss to follow‐up (*n* = 24; 20 from a single site that was closed in 2018 for reasons unrelated to the study), and physician's decision (*n* = 4). Of the 54 patients who discontinued, 26 underwent assessments for early termination.

Baseline demographics and disease characteristics (Table 1) were similar to those reported previously for all enrolled patients (*n* = 300).^10^ Briefly, patients had a mean age of 35.5 years and a mean AD disease duration of 26.6 years; 173 patients (60.1%) were male. Approximately 75% of patients had at least one type 2 inflammatory comorbidity at baseline. In terms of disease characteristics, patients had a mean of 9.8 visits for AD treatment in the past year, a mean EASI of 25.4, a mean peak pruritus NRS score of 6.5, and a mean DLQI of 8.3.

**TABLE 1:** Baseline demographics and clinical characteristics of patients

	All (*n* = 288)
Age (years)	35.5 ± 10.5
Male	173 (60.1)
Weight (kg)	62.0 ± 11.7
BMI (kg/m^2^)	22.7 ± 3.7
Age of AD onset (years)	9.0 ± 12.1
AD disease duration (years)	26.6 ± 12.1
Type 2 inflammatory comorbidities	217 (75.3)
Number of visits for AD treatment in the past 1 year^a^	9.8 ± 11.1
IGA score	3.3 ± 0.4
EASI	25.4 ± 15.5
BSA affected by AD (%)	50.9 ± 24.2
Peak pruritus NRS score^a^	6.5 ± 2.2
POEM score	16.8 ± 6.7
DLQI total score	8.3 ± 6.4

*Note:* Data are presented as mean ± SD or *n* (%).

Abbreviations: AD, atopic dermatitis; BMI, body mass index; BSA, body surface area; DLQI, Dermatology Life Quality Index; EASI, Eczema Area and Severity Index; IGA, Investigator's Global Assessment; NRS, numerical rating scale; POEM, Patient‐Oriented Eczema Measure; SD, standard deviation.

^a^ *n* = 286.

### 3.2 Treatment

At baseline, all but one patient received topical therapy, and all patients received topical treatment within the first year of observation. The most common baseline topical treatment was TCS of the second‐highest potency (“very strong”, 86.5% at baseline) followed by “medium” potency TCS (50.3% at baseline). Almost 60% of patients were treated with the highest potency TCS (“strongest”), and more than 60% were treated with TCI within the first year (Table 2). At baseline, 14.6% of patients were receiving at least one immunosuppressant, with the total proportions of patients receiving immunosuppressants increasing to 27.1% and 29.9% by months 12 and 24, respectively (Table 2). Of note, 13.5% and 18.8% of patients were treated at least once with oral corticosteroids or oral non‐steroidal systemic immunosuppressants (mostly cyclosporine A) during the 2‐year observation period. More than 10% of patients were treated with phototherapy, and more than 7% of patients were treated with Chinese herbal medicines (Kampo) during the 2‐year observation period. Antihistamines and antiallergic drugs (e.g., mediator release inhibitors) were widely prescribed (81.9% at baseline and 91.7% or more at ≥1 year). No biologics had been approved for AD in Japan until dupilumab was first marketed in April 2018, and only six patients were treated with biologics (most likely dupilumab) during the 2‐year observation period (Table 2).

**TABLE 2:** Summary of AD medications and therapies at baseline and from baseline to ~12 and ~24 months

Type of concomitant medication	Baseline (*n* = 288)	Baseline to ~12 months (*n* = 288)	Baseline to ~24 months (*n* = 288)
Topical			
Any TCS^a^ and/or TCI	287 (99.7)	288 (100.0)	288 (100.0)
TCS: strongest	120 (41.7)	167 (58.0)	177 (61.5)
TCS: very strong	249 (86.5)	270 (93.8)	277 (96.2)
TCS: strong	68 (23.6)	123 (42.7)	144 (50.0)
TCS: medium	145 (50.3)	186 (64.6)	194 (67.4)
TCS: weak	8 (2.8)	20 (6.9)	25 (8.7)
TCI	108 (37.5)	182 (64.6)	197 (68.4)
Topical only (excluding systemic or UV phototherapy)	231 (80.2)	182 (63.2)	172 (59.7)
Systemic anti‐inflammatory			
Any oral immunosuppressive therapy	42 (14.6)	78 (27.1)	86 (29.9)
Oral corticosteroids	12 (4.2)	33 (11.5)	39 (13.5)
Oral non‐steroidal immunosuppressants^b^	31 (10.8)	51 (17.7)	54 (18.8)
Biologics^c^	0	0	6 (2.1)
UV phototherapy	16 (5.6)	33 (11.5)	35 (12.2)
Adjunctive			
Antihistamines/anti‐allergic drugs^d^	236 (81.9)	264 (91.7)	267 (92.7)
Chinese herbal medicine	6 (2.1)	16 (5.6)	21 (7.3)
Psychotherapy	0	4 (1.4)	4 (1.4)

*Note:* Data are presented as *n* (%).

Abbreviations: AD, atopic dermatitis; TCI, topical calcineurin inhibitors; TCS, topical corticosteroids; UV, ultraviolet.

^a^ TCS rank classification is different in Japan compared with Europe or the USA and TCS are generally classified into five ranks: strongest (e.g., 0.05% clobetasol propionate), very strong (e.g., 0.1% mometasone furoate), strong (e.g., 0.3% deprodone propionate), medium (e.g., 0.3% prednisolone valerate acetate), and weak (e.g., 0.5% prednisolone).

^b^ Only class name was collected in the study; the only licensed oral non‐steroidal immunosuppressant for AD in Japan is cyclosporine A.

^c^ No biologics were approved for AD in Japan at the start of this study; dupilumab was the only biologic approved for AD during the study period.

^d^ Anti‐allergic drugs included thromboxane A2 inhibitors, leukotriene receptor antagonists, and cytokine inhibitors (suplatast tosilate).

### 3.3 Treatment effectiveness

#### 3.3.1 Investigator's Global Assessment

**FIGURE 1:**
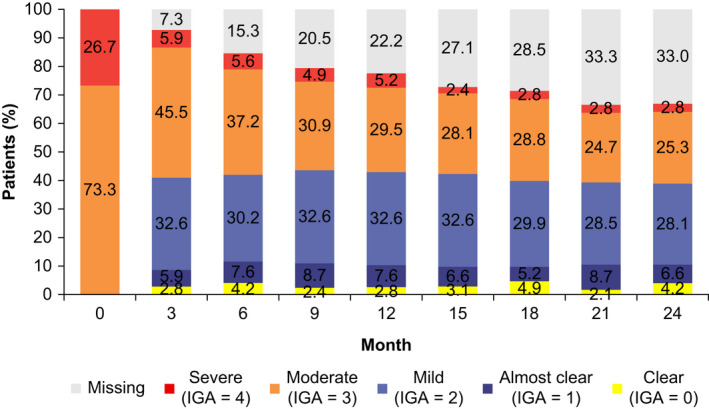
Stacked bar chart of longitudinal IGA score. IGA, Investigator's global assessment.

Post‐baseline, disease severity in most patients improved within the first 3 months, and the proportions of patients with an IGA score of 0 (clear), 1 (almost clear), and 2 (mild) remained at the same levels from then until the end of the study at month 24 (Figure 1). The proportions of patients with scores of 3 (moderate) and 4 (severe) decreased gradually over time; however, 28.1% of enrolled patients had IGA scores of 3 or 4 at the end of the study (Figure 1). At month 3 and thereon, higher proportions of patients achieved IGA scores corresponding to “clear” or “almost clear” in the group with baseline IGA score of 4 (severe) compared with those with an IGA score of 3 (moderate) (Figure S1). Totals of 10.8% and 13.9% of patients had reached an IGA score corresponding to “clear” and “almost clear” at any point during the entire study period, respectively, while 47.9% attained a minimum IGA score corresponding to “mild” (Table 3).

**TABLE 3:** Minimum IGA score during the study period

Minimum IGA	All (*n* = 288)	Baseline IGA score	
		3 (moderate) (*n* = 211)	4 (severe) (*n* = 77)
0	31 (10.8)	18 (8.5)	13 (16.9)
1	40 (13.9)	26 (12.3)	14 (18.2)
2	138 (47.9)	113 (53.6)	25 (32.5)
3	71 (24.7)	54 (25.6)	17 (22.1)
4	8 (2.8)	0	8 (10.4)

*Note:* Data are presented as *n* (%).

Abbreviation: IGA, Investigator's Global Assessment.

The six patients who were treated with a biologic (assumed dupilumab) at month 15 or later had IGA scores of 3 (moderate) before the biologic was initiated and all but one had IGA scores of 1 (almost clear) or 2 (mild) at the end of the study (Figure S2).

#### 3.3.2 Flares

**FIGURE 2:**
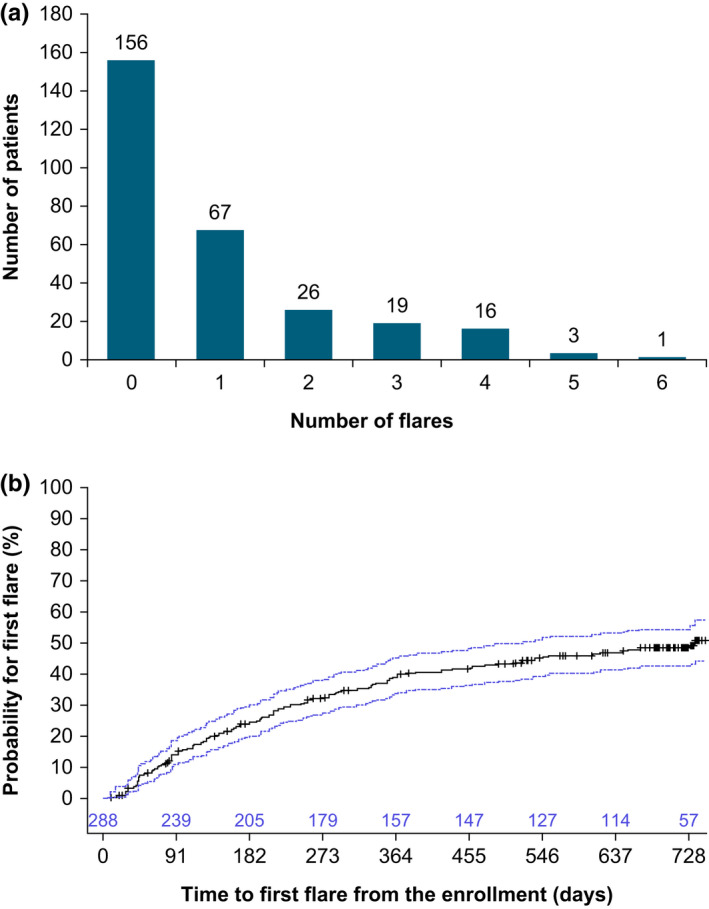
(a) Number of patients categorized by number of flares during the study (overall, in 0–24 months). (b) Kaplan–Meier analysis of time to first flare occurrence. Blue lines signify 95% confidence intervals; crosses show right‐censoring.

A total of 132 patients (45.8%) had at least one flare, and 65 (22.6%) patients had ≥2 flares over 2 years (Figure 2a). The Kaplan–Meier analysis of time to first flare showed that approximately 40% of patients had their first flare within the first year of the study, with the probability of developing flares increasing to 50% by the end of the second year (Figure 2b). This is supported by an analysis of annualized flares, which showed that the maximum rate was 0.97 flares/patient‐year during the first 3 months, then decreased over time to the end of the study, reaching an overall 0.53 flares/patient‐year during the 24 months (Table S2). The most common investigator‐reported reasons for flares were environmental (e.g., particular allergen exposure) (24.9%), sweating (20.7%), and poor treatment adherence (16.1%, Figure S3).

#### 3.3.3 Other measures of effectiveness

**FIGURE 3:**
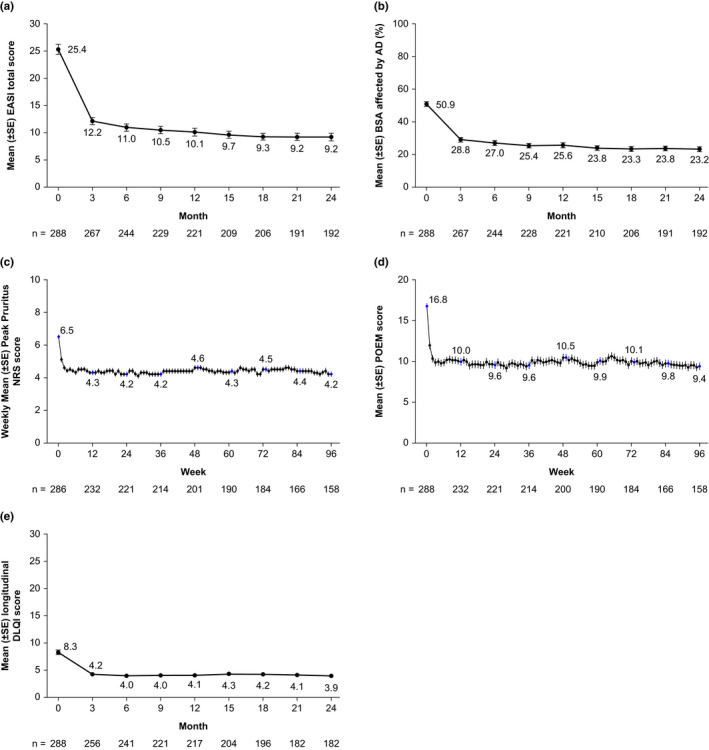
Longitudinal scores (mean ± SE) in the overall population over time: (a) EASI; (b) percent BSA affected by AD; (c) peak pruritus NRS; (d) POEM; and (e) DLQI. AD, atopic dermatitis; BSA, body surface area; DLQI, Dermatology Life Quality Index; EASI, Eczema Area and Severity Index; NRS, numerical rating scale; POEM, Patient‐Oriented Eczema Measure; SE, standard error.

In the overall population, mean EASI and BSA affected by AD improved by approximately 50% from baseline to month 3, with gradual incremental improvement seen through month 24 (Figure 3a,b). The highest degree of improvement was seen in the subgroups of patients who were receiving topical treatment only or non‐steroidal oral immunosuppressants at baseline (Figure S4a–c).

Similarly to the assessment of signs, the majority of improvement in itch (assessed by weekly peak pruritus NRS scores) and symptoms (assessed by weekly POEM scores) occurred early within the first 2 weeks of the study (Figure 3c,d, Figure S4d,e). Furthermore, DLQI, which measures quality of life, also showed a decrease at month 3, after which values were maintained during the study period (Figure 3e, Figure S4f). Finally, the proportions of patients who achieved a ≥4‐point improvement at month 24 from baseline in peak pruritus NRS, DLQI, and POEM were 34.5%, 54.1%, and 62.3%, respectively (Figure S5a–c).

### 3.4 Safety

Twenty‐one patients (7.3%) reported AE potentially related to treatment, of which 4.2% had infections and infestations (Table 4). Very few patients (1.0%) had treatment discontinuations due to AE. Skin infections requiring treatment were reported in 48 patients (16.7%), indicative of inadequate disease control. Finally, serious AE were reported in five patients (1.7%), of which only one event of erysipelas was reported as related to treatment (skin moisturizer) by the investigator.

**TABLE 4:** Collected safety results^a^

	All (*n* = 288)
AE potentially related to AD treatment	21 (7.3)
Infections and infestations^b^	12 (4.2)
AE leading to AD treatment discontinuation	3 (1.0)
Infection	3 (1.0)
Skin infection requiring treatment reported as AE	48 (16.7)
Serious AE	5 (1.7)

*Note:* Data are presented as *n* (%).

Abbreviations: AD, atopic dermatitis; AE, adverse event.

^a^ Summary of patients with ≥1 AE potentially related to AD treatment, AE leading to AD treatment discontinuation, AE classified as skin infections requiring treatment, and serious AE.

^b^ Folliculitis (*n* = 3), erysipelas (*n* = 2), herpes simplex (*n* = 2), skin bacterial infection (*n* = 2), cellulitis (*n* = 1), herpes zoster (*n* = 1), Kaposi's varicelliform eruption (*n* = 1), and tinea infection (*n* = 1).

### 3.5 Biomarker analysis

Baseline values for serum TARC levels, peripheral blood eosinophil count, serum total IgE, and serum LDH levels were available for 67, 52, 66, and 71 patients, respectively, which are shown in Table S3, with median values showing marked increases with clear association with disease severity assessed by IGA; namely, patients with baseline IGA score of 4 (severe) showed greater increases than those with IGA score of 3 (moderate). Mean TARC, eosinophil, and LDH levels decreased rapidly from baseline to month 3, then remained at stable levels, while total IgE levels oscillated over time (Figure S6a–d).

## 4 DISCUSSION

Data from the ADDRESS‐J registry revealed the limited effectiveness of conventional therapies for Japanese adults with moderate‐to‐severe AD. A group of patients did not achieve adequate disease control, showing that the conventional standard treatment was insufficient in these patients. Furthermore, clinical response plateaued with topical treatments after an initial improvement within the first 3 months of the study.

For the purpose of this analysis, we used several measures of AD disease severity and quality of life impact, including clinician‐reported (objective) outcomes and PRO (subjective), as recommended by Japanese guidelines^5^ and the Harmonizing Outcomes Measures in Eczema (HOME) initiative.^18^ Although most assessments, including those of signs (IGA, EASI, and BSA), symptoms (peak pruritus NRS score and POEM), and quality of life (DLQI), showed improvement in AD severity over time, regardless of received treatment, high proportions of patients did not achieve sufficient long‐term disease control (annualized rates of flares). Severe disease burden, in terms of IGA scores of 3 (moderate) or 4 (severe), persisted in approximately 42% of observed patients despite long‐term (2‐year) management with TCS, TCI, adjunctive antihistamines/anti‐allergic drugs, oral systemic immunosuppressants, and UV phototherapy. Only 24.7% achieved minimum IGA scores of 0 (clear) or 1 (almost clear), and nearly half of the patients remained at a minimum score of 2 at any timepoint during the study. However, it should be noted that 54 of the enrolled patients discontinued early from the study (mostly due to consent withdrawal and loss to follow‐up), which could limit the interpretation of these data. The six patients who received a biologic during the study all had IGA scores of 3 at the time of biologic initiation (after ≥15 months of other treatments), and two patients achieved IGA scores of 1 after biologic treatment, indicating that such novel therapies might be alternatively efficacious in a subset of patients whose AD is inappropriately controlled with conventional medications and/or therapies.

Mean EASI scores decreased from 25.4 at baseline to 9.2 at the end of the study, while 45.3% of patients achieved EASI‐75 (i.e., a ≥75% improvement from baseline in EASI) at the end of the study. According to the reported severity strata for EASI,^19^ scores of 25.4 and 9.2 correspond to severe and moderate, respectively. Peak pruritus NRS scores decreased from 6.5 at baseline to 4.2 at the end of the study, corresponding to moderate pruritus.^20^ Only 34.5% of patients achieved ≥4‐point improvement from baseline in peak pruritus NRS (scale 0–10) at the end of the study, while 54.1% and 62.3% of patients achieved ≥4‐point improvements from baseline in DLQI and POEM (scales 0–30), respectively (Figure S5). Of note, the minimal clinically important difference for improvement in DLQI and POEM scores are reported as 4.0 and 3.4, respectively,^21^, ^22^ highlighting that significant proportions of patients did not achieve clinically meaningful response with conventional treatments.

There was a tendency for patients who were only receiving topical treatment at baseline to show an apparent numerically higher improvement in terms of AD signs during follow‐up compared with patients who were receiving phototherapy or oral corticosteroids at baseline. However, due to the low numbers of patients using treatments other than topical medications and the fact that subsequent treatments were not taken into account (subgroups were defined based on baseline treatment), these results should be interpreted with caution.

In Japan, topical therapy and antihistamines/anti‐allergic drugs are widely used for the management of AD, while systemic therapies (such as oral immunosuppressants) and phototherapy are limitedly used when assessed at one time point (e.g., baseline). However, in the 2‐year observation of this cohort, nearly 30% of patients were treated with oral immunosuppressants and 12% were treated with phototherapy. This indicates that long‐term disease control is not easily achieved with topical treatments in some patients. AD is a dynamic chronic disease characterized by relapses, with the severity and frequency of flares as a surrogate marker for control. In the current study, 65 patients (22.6%) had multiple flares over the entire observation period. This shows that there is a need for expanded systemic treatment options. Nonetheless, IGA scores of 0, 1, or 2, or EASI‐75 were achieved by a certain subset of patients. Factors associated with response in these patients will be explored in future analyses.

The biomarker analyses showed rapid and sustained decreases in mean serum TARC levels, which has been found to be a very reliable biomarker for AD severity.^5^, ^23^ Peripheral blood eosinophil count and serum LDH also decreased by month 3 and remained reduced, and these have also been shown to correlate well with AD severity.^23^ However, mean serum total IgE levels varied during the course of the study, with high interpatient variability, which is in line with previous studies that have shown poor correlations between IgE and AD severity and high interpatient variability of IgE.^24^ Nevertheless, biphasic changes in total IgE levels over the 2 years may suggest some seasonal factors for this biomarker.

The strength of this analysis lies in the fact that it is based on a recent real‐world study that describes treatment patterns, effectiveness, and safety in Japanese patients with moderate‐to‐severe AD. However, this analysis also has several limitations. One limitation is the decreasing number of patient assessments at later timepoints with unknown reasons, while another is that the reasons for missing data were not recorded for some patients. Furthermore, due to the inclusion criteria (age 20–59 years; diagnosis of AD ≥6 months previously; IGA score ≥3; with treatment escalation at the baseline visit; and willingness to participate in the study for 2 years), these results might not be reflective of all adults with AD in Japan. We also noted that patients enrolled at different sites had varying levels of severity and adherence, so results may not be applicable to all treatment facilities. Lastly, while the six patients who initiated biologics during the study had improvements in IGA, there were too few patients to draw any firm conclusions.

In the ADDRESS‐J study of Japanese adults with moderate‐to‐severe AD, the disease severity assessed by IGA improved in some, but did not improve in a substantial proportion of patients, and flares were common despite long‐term management with TCS and/or TCI, adjunctive antihistamines/anti‐allergic drugs, systemic immunosuppressants, and UV phototherapy. Furthermore, outcome measures relating to symptoms and quality of life showed a plateauing of response and failed to demonstrate clinically meaningful changes in many patients. The emergence of newly approved systemic agents, such as biologics, may provide a potential strategy for the long‐term management of a subset of patients with inadequately controlled moderate‐to‐severe AD in such a population.

## CONFLICT OF INTEREST

N. Katoh has received honoraria for lectures from AbbVie, Celgene Japan, Eli Lilly Japan, Janssen Pharma, Kyowa Kirin, Leo Pharma, Maruho, Mitsubishi Tanabe Pharma, Sanofi, and Taiho Pharma; research grants from AbbVie, Eli Lilly Japan, Kyowa Kirin, LEO Pharma, Maruho, Mitsubishi Tanabe Pharma, Sanofi, Sun Pharma, and Taiho Pharmaceutical. H. Saeki reports honoraria for lectures from AbbVie, Kyorin Pharmaceutical, Kyowa Kirin, Maruho, Mitsubishi Tanabe Pharma, Sanofi, and Taiho Pharma; and research grants from Eisai, Tokiwa Pharmaceutical, and Torii Pharmaceutical. Y. Kataoka reports honoraria for lectures and contract research grants from Sanofi, and research grants from AbbVie, Eli Lilly, LEO Pharma, Maruho, Otsuka, and Pfizer. T. Etoh has received honoraria for lectures from Kyowa Hakko Kirin and Maruho. S. Teramukai has received honoraria for lectures from Bayer, Chugai Pharmaceutical; a research grant from Nippon Boehringer Ingelheim; and consultant fees from Daiichi Sankyo, Gunze, Sanofi, Solasia Pharma, Sysmex, and Takeda. H. Takagi, H. Fujita, and K. Arima are Sanofi K.K. employees and may hold stock and/or stock options in the company. M. Ardeleanu is an employee and shareholder of Regeneron Pharmaceuticals, Inc. E. Rizova was an employee of Sanofi and might hold stock and/or stock options in the company when the study was conducted.

## Supporting information


Appendix S1
