# Supplementary material for: Evaluation of standard treatments for managing adult Japanese patients with inadequately controlled moderate‐to‐severe atopic dermatitis: Two‐year data from the ADDRESS‐J disease registry
Source: J Dermatol. 2022 Jun 17;49(9):903–11. doi: 10.1111/1346-8138.16485 (PMC9543354; doi:10.1111/1346-8138.16485)
Supplement: Supplementary file 1 — Appendix S1 [file JDE-49-903-s001.docx]

**Supporting information**

**TABLE S1** ADDRESS-J study investigators

| **Investigator** | **Affiliation** |
| --- | --- |
| Norito Katoh | Department of Dermatology, Kyoto Prefectural University of Medicine |
| Hidehisa Saeki | Department of Dermatology, Nippon Medical School |
| Yoko Kataoka | Department of Dermatology, Osaka Habikino Medical Center |
| Takafumi Etoh | Department of Dermatology, Tokyo Teishin Hospital |
| Shun'ichi Sawada | Sawada Dermatology Clinic |
| Minako Yasumoto | Mildix Skin Clinic |
| Yoshiyuki Murakami | Mildix Skin Clinic, Yokohama Medical Center |
| Tomoki Okuda | Okuda Dermatology Clinic |
| Shiomi Kawano | Iidabashi Skin Clinic |
| Yoko Todoroki | Clinique Dermatologique Todoroki |
| Tokuya Omi | Queen's Square Medical Center |
| Yusei Itoh | Itoh Skin Clinic |
| Ryuji Maruyama | Maruyama Hifuka Clinic |
| Hiroshi Ikeda | Kohoku Dermatology |
| Masaru Igarashi | Igarashi Dermatology Clinic |
| Katsumi Tanito | Tsukuda River-City Dermatology Clinic |
| Toshiya Asai | Asai Dermatology Clinic |
| Mineko Suzuki | M&M Skincare Clinic |
| Emi Nakazaki | emi Skin Clinic Shoto |
| Miwako Kinoshita | Kinoshita Skin Clinic |
| Haruko Mizutani | Mizutani Dermatology Clinic |
| Kazutoshi Hamada | Hamada Clinic |
| Toshiya Ebata | Chitofuna Dermatology Clinic |
| Michihiro Hide | Department of Dermatology, Graduate School of Biomedical and Health Sciences, Hiroshima University |
| Junichi Sugai | Sugai Dermatology Park Side Clinic |
| Naoko Ishiguro | Department of Dermatology, Tokyo Women's Medical University |
| Tomomitsu Miyagaki | Department of Dermatology, The University of Tokyo Graduate School of Medicine |
| Saori Itoi-Ochi | Department of Dermatology, Osaka University Graduate School of Medicine |
| Hiroshi Mitsui | Department of Dermatology, Faculty of Medicine, University of Yamanashi |
| Kazumoto Katagiri | Department of Dermatology, Dokkyo Medical University Saitama Medical Center |

**Table S2.** Incidence rate of flares assessed every 3 months.

|  | **0–3 months** | **3–6 months** | **6–9 months** | **9–12 months** | **12–15 months** | **15–18 months** | **18–21 months** | **21–24 months** | **Overall** |
| --- | --- | --- | --- | --- | --- | --- | --- | --- | --- |
| Number of patients | 288 | 272 | 266 | 254 | 246 | 238 | 219 | 205 | 288 |
| Total number of flares | 71 | 38 | 33 | 34 | 28 | 23 | 18 | 16 | 261 |
| Total years at risk† | 73.54 | 68.90 | 63.92 | 63.22 | 60.61 | 57.72 | 56.76 | 48.78 | 493.45 |
| Incidence rate (95% CI), flares/PY | 0.97 (0.75–1.22) | 0.55 (0.39–0.76) | 0.52 (0.36–0.73) | 0.54 (0.37–0.75) | 0.46 (0.31–0.67) | 0.40 (0.25–0.60) | 0.32 (0.19–0.50) | 0.33 (0.19–0.53) | 0.53 (0.47–0.60) |

Abbreviations: CI, confidence interval; PY, patient-years.

†Years at risk was calculated as follows: Years at risk = ([Date of visit] – [Date of enrollment] + 1) / 365.25. Each data point was set at 3 months, but with 1.5 months as an allowance, so, for example, the 0–3 months patient data include patients up to 3–4.5 months.

**TABLE S3** Biomarker analysis in selected patients at baseline

|  | **n** | **All patients** | **Baseline IGA score** | | | |
| --- | --- | --- | --- | --- | --- | --- |
|  |  |  | **n** | **3 (moderate)** | **n** | **4 (severe)** |
| Serum TARC (pg/mL) | 67 | 3,157  (331–58,677) | 26 | 1465  (331–25,750) | 41 | 3,870  (450–58,677) |
| Peripheral blood eosinophil count (Giga/L) | 52 | 839  (110–3,540) | 28 | 618  (110–2,780) | 24 | 1062  (520–3,540) |
| Serum IgE (IU/mL) | 66 | 6,289  (45–66,200) | 27 | 1,908  (59–28,587) | 39 | 8772  (45–66,200) |
| Serum LDH (IU/L) | 71 | 293  (125–858) | 29 | 247 (125–422) | 42 | 316 (193–858) |

*Note:* Data are presented as median (minimum–maximum).

IgE, immunoglobulin E; LDH, lactate dehydrogenase; TARC, thymus and activation-regulated chemokine.

**FIGURE S1** Stacked bar chart of longitudinal IGA score by baseline IGA score.


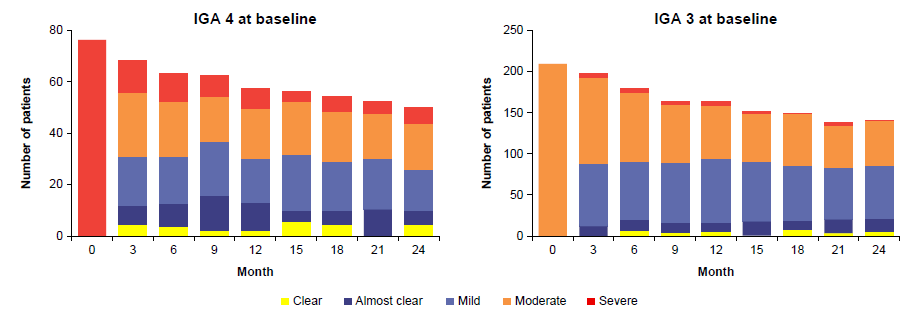


Abbreviation: IGA, Investigator’s Global Assessment.

**FIGURE S2** IGA scores among the 6 patients who initiated a biologic (assumed dupilumab) during the study. The information below the bars indicates when each patient started the biologic.

Abbreviation: IGA, Investigator’s Global Assessment.

**FIGURE S3** Reported reason for flares (n = 261).

**FIGURE S4** Longitudinal scores (mean ± SE) by baseline medication over time: (a) EASI; (B) EASI-75; (c) percent BSA affected by AD; (d) peak pruritus NRS; (e) POEM; (f) DLQI.

Abbreviations: AD, atopic dermatitis; BSA, body surface area; DLQI, Dermatology Life Quality Index; EASI, Eczema Area and Severity Index; EASI-75, ≥75% improvement from baseline in EASI; NRS, numerical rating scale; OCS, oral corticosteroids; POEM, Patient-Oriented Eczema Measure; SE, standard error.


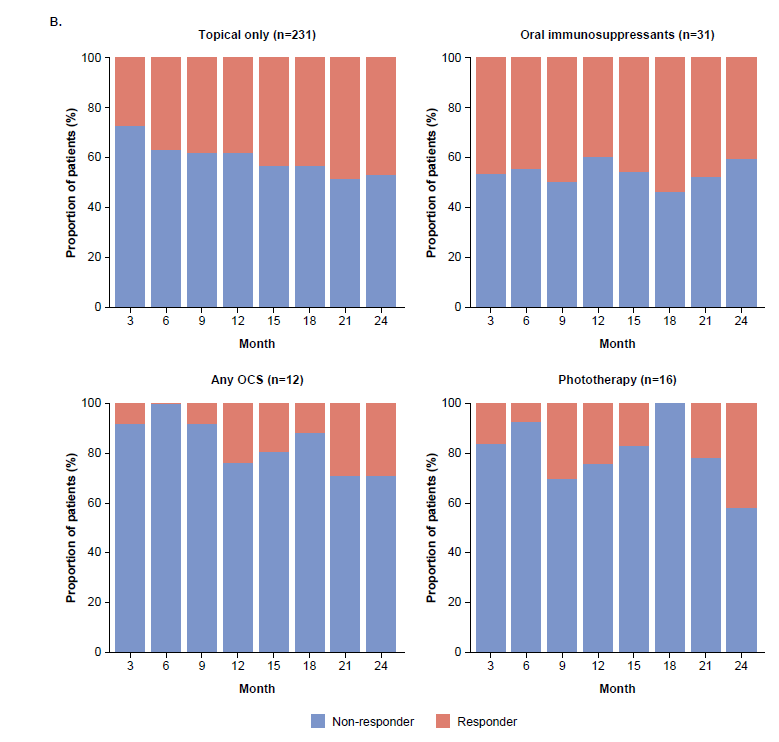

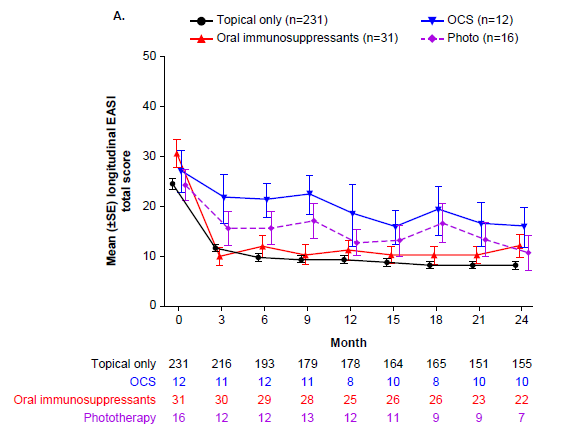


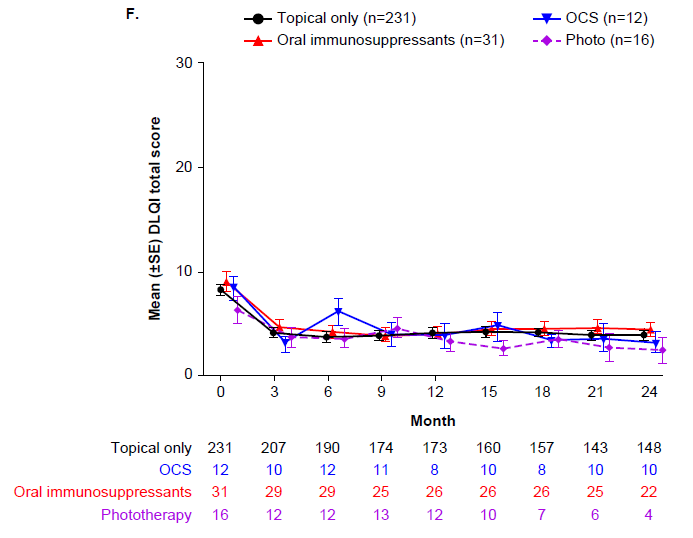

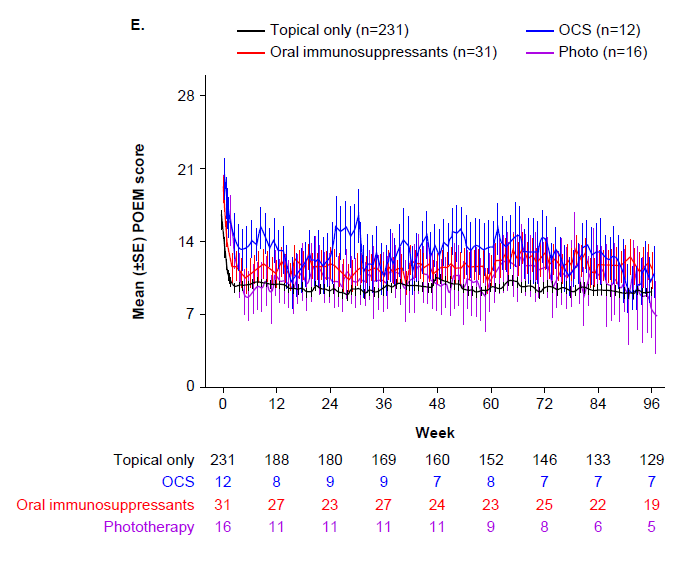

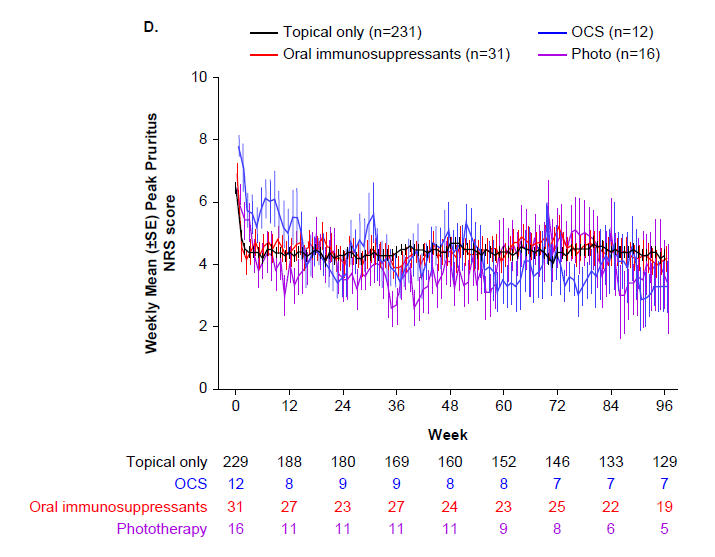

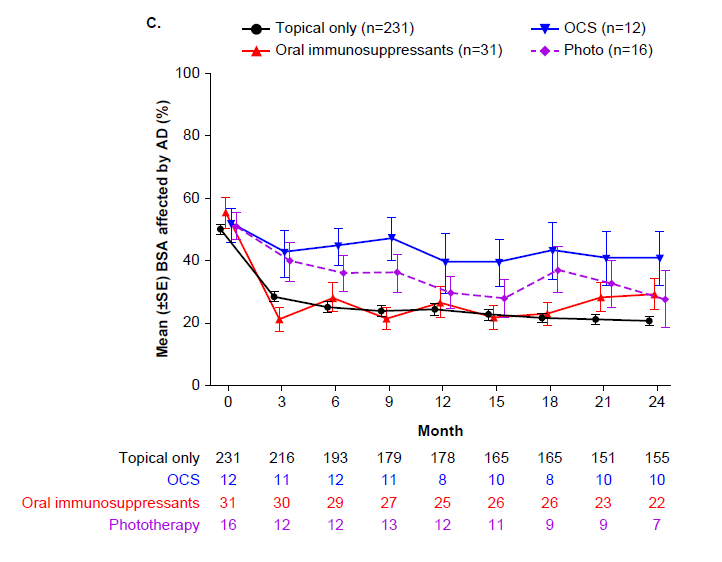


**FIGURE S5** Proportions of patients with ≥4-point improvement from baseline in (a) peak pruritus NRS; (b) DLQI; and (c) POEM.


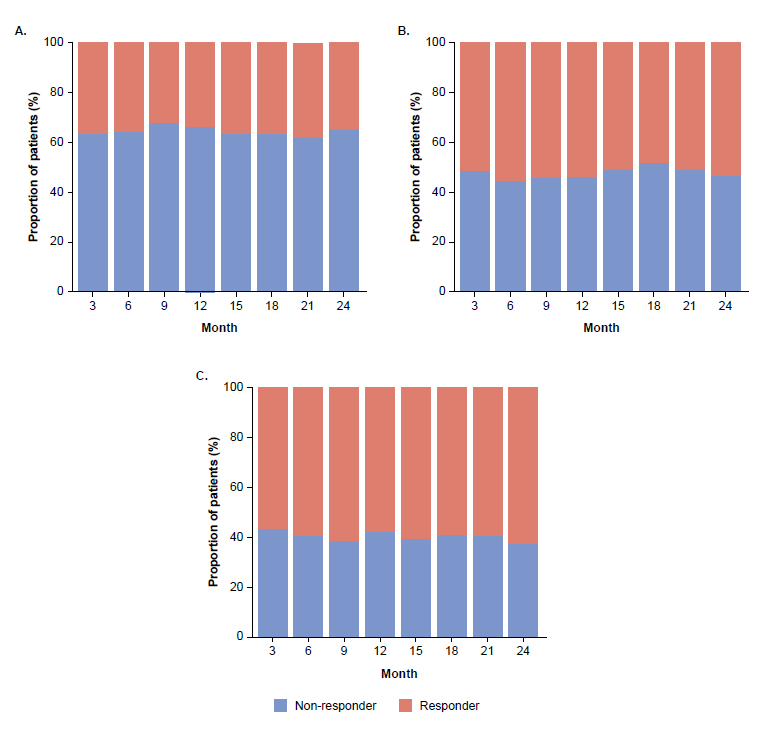


Abbreviations: DLQI, Dermatology Life Quality Index; NRS, numerical rating scale; POEM, Patient-Oriented Eczema Measure.

**FIGURE S6** Longitudinal assessment of biomarkers (mean [SE]): (a) serum TARC; (b) peripheral blood eosinophil count; (c) serum total IgE; and (d) serum LDH.


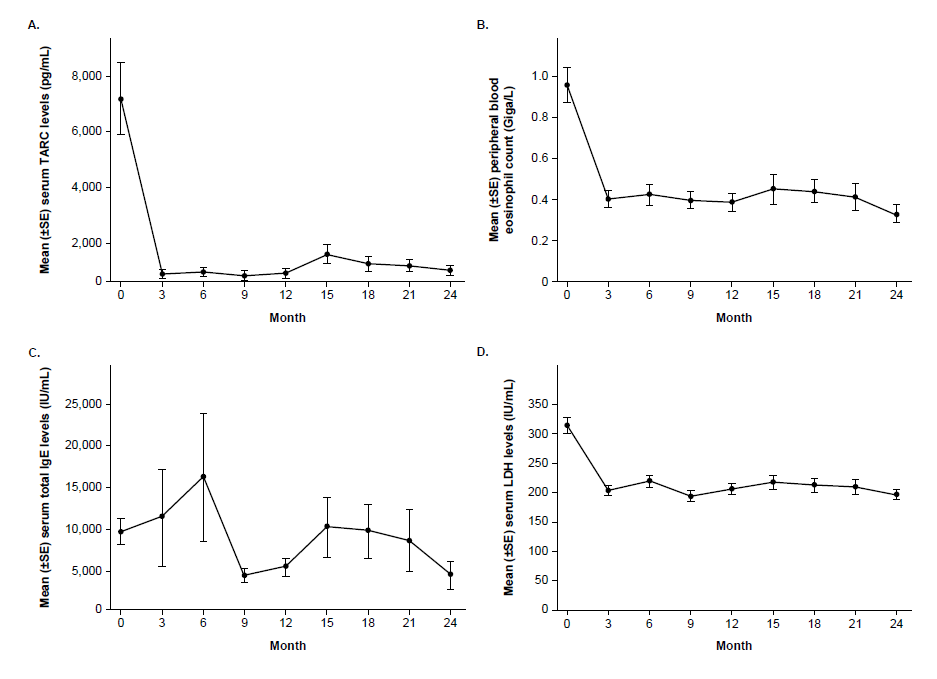


Abbreviations: IgE, immunoglobulin E; LDH, lactate dehydrogenase; SE, standard error; TARC, thymus and activation-regulated chemokine.
